# Distal defect of the humerus, a possible normal variant: a case report

**DOI:** 10.1186/s13256-017-1395-z

**Published:** 2017-08-17

**Authors:** Akio Sakamoto, Shuichi Matsuda

**Affiliations:** Department of Orthopaedic Surgery, Graduate School of Medicine, Kyoto University, Shogoin, Kawahara-cho 54, Sakyo-ku, Kyoto, 606-8507 Japan

**Keywords:** Humerus, Normal variant, MRI, Case report

## Abstract

**Background:**

Many normal variants of bones on plain radiographs have been reported.

**Case presentation:**

In the current report, a 14-year-old Asian girl noticed an occasional slight elbow pain. She had no traumatic episode. Plain radiographs showed a well-defined osteolytic lesion with a sclerotic rim, which was continuous with the normal subarticular bone in the distal humerus. Magnetic resonance imaging revealed that the defect area seen on the plain radiograph showed low-signal to iso-signal intensity on T1-weighted images and slightly high-signal intensity on T2-weighted fat suppression images. Bone edema was not observed. The association between her elbow pain and the lesion was not conclusive.

**Conclusions:**

The findings from the images suggested that the lesion was a normal variant rather than osteochondritis dissecans or a neoplastic lesion, and possibly an anatomical counterpart of a dorsal defect of the patella.

## Background

Normal variants can be defined for lesions with atypical findings, and are normally found in some percentage of the population. Normal skeletal variants identified from plain radiographs have been listed in the well-known book *Atlas of Normal Roentgen Variants That May Simulate Disease* [1].

The current report describes a characteristic osteolytic lesion with a clear osteosclerotic rim at the distal humerus. This lesion, previously unreported, may be an anatomical counterpart of a dorsal defect of the patella. A distal defect of the humerus is a normal variant, and the discussion about the current case was made in light of a dorsal defect of the patella [2].

## Case presentation

**Fig. 1:**
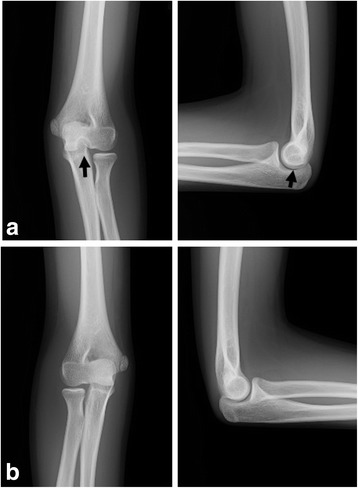
A 14-year-old girl with distal defect of the humerus. Plain radiographs show a well-defined osteolytic lesion with sclerotic rim on the left distal humerus (black arrows). Note that the osteosclerotic rim is absent at the joint periphery (**a**). The right side of the distal humerus is normal (**b**)

A 14-year-old Asian girl had no pain in daily life, but occasionally had a slight pain in her left elbow. She looked normally developed, and was not obese. There was no past medical, surgical, or family history of factors that might contribute to bone disease. She had no tenderness over the elbow. She was not an active athlete, but belonged to a basketball club in school. There was no history of trauma. Plain radiographs showed a well-defined osteolytic lesion with an osteosclerotic rim in the distal humerus. The osteosclerotic rim was absent at the joint periphery, but continued to the subarticular bone. The osteosclerotic rim had no irregularity. Periosteal reaction was not seen. Her right elbow was normal (Fig. 1).

**Fig. 2:**
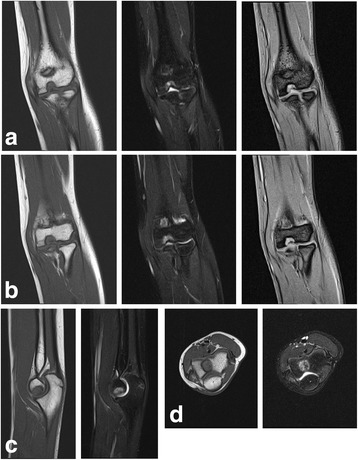
A 14-year-old girl with distal defect of the humerus. Coronal magnetic resonance imaging shows low-signal to iso-signal intensity on T1-weighted images (*left*) and slightly high-signal intensity on T2-weighted fat suppression (*middle*) and short tau inversion recovery images (*right*) (**a** and **b**, sequential sections). Sagittal (**c**) and coronal (**d**) sections are present (*left*, T1-weighted image; *right*, T2-weighted image)

Magnetic resonance imaging (MRI) revealed that the osteolytic lesion was solid, and was next to the surrounding joint cartilage. The lesion had low-signal to iso-signal intensity on T1-weighted images, slightly high-signal intensity on T2-weighted fat suppression images, and high-signal intensity on short tau inversion recovery (STIR) images (Fig. 2). Bone marrow edema of low-signal intensity on T1-weighted images and high-signal intensity on T2-weighted images was not seen.

The osteolytic lesion had a differential diagnosis of osteochondrosis dissecans as an osteocartilaginous defect. In the current case, the osteolytic area was rather deep and the osteosclerotic rim was clear. Our patient had no traumatic episode, and a clinical symptom of rocking at the elbow was not observed. No free body was detected in her elbow joint space on MRI. These image and clinical findings did not meet the diagnostic criteria for osteochondrosis dissecans.

A neoplastic lesion was also a differential diagnosis in the current case. Osteolytic bone tumors that involve the epiphysis include chondroblastoma, chondromyxoid fibroma, and giant cell tumor of bone. An osteosclerotic rim is not usually seen in giant cell tumor of bone. Although chondroblastoma and chondromyxoid fibroma may have an osteosclerotic rim, the osteosclerotic rim would not be as clear as that in the current case. Furthermore, the absence of bone marrow edema suggested a lower probability of a neoplastic lesion.

After excluding the possible differential diagnoses of traumatic and neoplastic lesions, a normal variant condition was considered. Biopsy was not performed for the diagnosis. The plain radiograph at follow-up at 2 years had not changed, and pain was not noticed at that time.

## Discussion

**Fig. 3:**
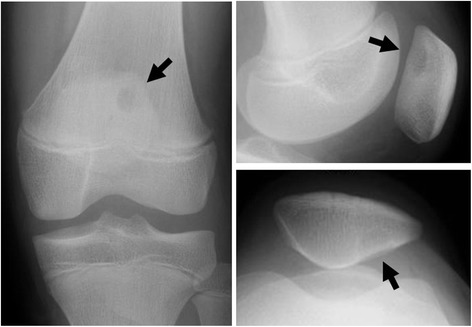
An 11-year-old girl with a dorsal defect of the patella. Plain radiographs show a well-defined osteolytic lesion with a sclerotic rim on the dorsal patella (black arrows)

The current lesion of a so-called distal defect of the humerus was considered to be a normal variant, but this variant is not listed in the text book [1]. The plain radiographic appearance of the current case is characteristic, and was reminiscent of a dorsal defect of the patella (Fig. 3) [3, 4]. The dorsal defect of the patella is located in the superolateral part of the patella [4, 5]. The incidence of dorsal defect of the patella is 0.3 to 1% [6]. Unilateral and bilateral cases are reported [7]. The etiology of the dorsal defect of the patella is unknown, and its differential diagnosis is osteochondritis dissecans, as well as neoplastic lesion [8].

The diagnosis of dorsal defect of the patella can be made from a plain radiograph, and invasive diagnostic procedures including a biopsy should be avoided [3]. In the current report, the diagnosis of distal defect of the humerus was made in light of the dorsal defect of the patella. Because there was no bone marrow edema in MRI, the lesion was unlikely to be a neoplastic lesion. Therefore, the diagnosis was made based on the image, and a biopsy was judged to be unnecessary.

Most cases with dorsal defect of the patella cause no pain, and the lesions are usually found incidentally. However, dorsal defect of the patella causing knee pain has been reported [9–11]. In the current case, the absence of objective findings over the elbow and of bone marrow edema made it less likely as the cause of the elbow pain. Furthermore, the elbow pain spontaneously disappeared. However, the absence of association is not conclusive, and the current occasional elbow pain might be associated with the lesion, taking into consideration cases of a painful dorsal defect of the patella.

## Conclusions

The characteristic imaging findings at the distal humerus suggest that this lesion is a normal variant and possibly an anatomical counterpart of dorsal defect of the patella.
